# Supplementary material for: Optimal percentage of inhibitory synapses in multi-task learning
Source: Sci Rep. 2015 Apr 22;5:9895. doi: 10.1038/srep09895 (PMC4405698; doi:10.1038/srep09895)
Supplement: Supplementary Information — Supplementary Material [file srep09895-s1.pdf]

Optimal percentage of inhibitory synapses in multi-task learning

Vittorio Capano

Physics Department, University of Naples Federico II, Napoli, Italy

Hans J. Herrmann

Institute Computational Physics for Engineering Materials,

ETH, Zürich, CH; Departamento de Física,

Universidade Federal do Ceará, 60451-970 Fortaleza, Ceará, Brazil

Lucilla de Arcangelis

Department of Industrial and Information Engineering,

Second University of Naples and INFN Gr. Coll. Salerno, Aversa (CE), Italy

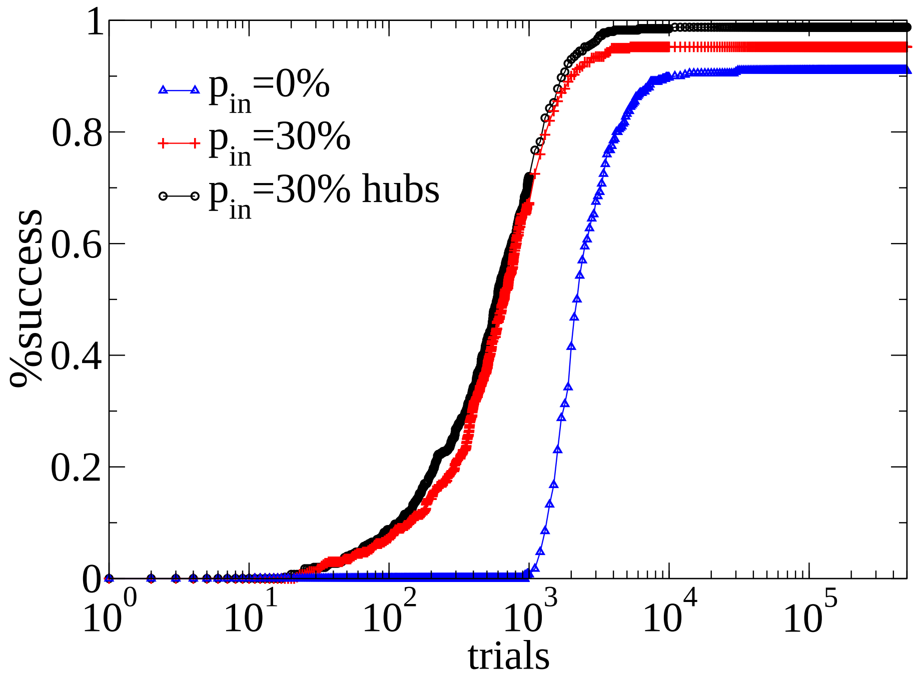

Supplementary Figure 1. Percentage of networks giving the right answer to the XOR rule as a function of the number of times the rule is applied for 500 configurations with $N = 250$ neurons ($k_d = 3$, $\alpha = 0.001$, homeostatic plasticity). Three different cases are analysed: purely excitatory networks, networks with $p_{in} = 30\%$ inhibitory synapses with random connectivity degree and networks where inhibitory synapses are assigned to random neurons with $k_{out} > 10$. The best performance is obtained for inhibitory neurons highly connected also for different plastic adaptations. The same behaviour is observed also for other rules and other values of $p_{in} > 0$.

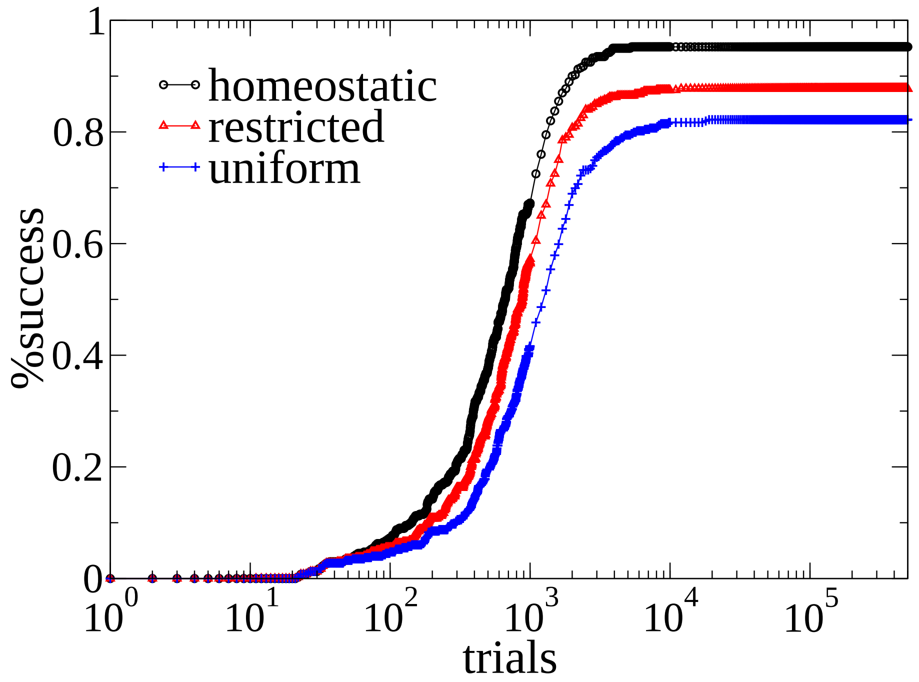

Supplementary Figure 2. Percentage of networks giving the right answer to the XOR rule as a function of the number of times the rule is applied for 500 configurations with $N = 250$ neurons ($k_d = 3$, $\alpha = 0.001$, $p_{in} = 0.3$ no hubs). Three different plastic adaptations are analysed: Uniform (all active synapses undergo the same modification independently of their excitatory/inhibitor character), restricted (only excitatory synapses are modified), homeostatic (excitatory and inhibitory synapses undergo modifications with opposite sign). The best performance is obtained for homeostatic plasticity, also for different p_{in} and hub inhibitory neurons. The same behaviour is observed also for the other rules.

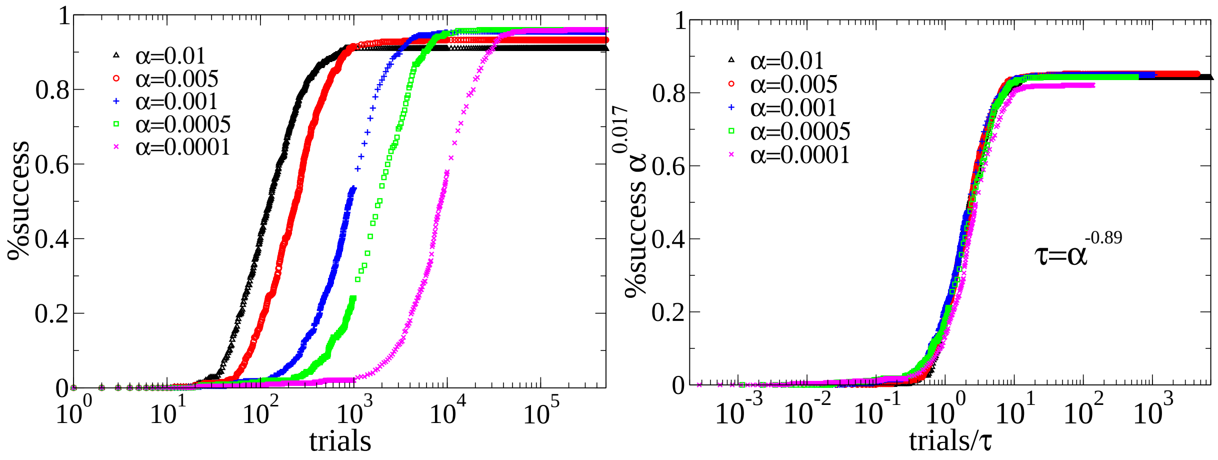

Supplementary Figure 3. Left: Percentage of networks giving the right answer to the parallel learning of XOR and AND rules as a function of the number of times the rule is applied for 500 configurations of networks with $N = 250$ neurons and different α ($k_d = 3$, $p_{in} = 0.3$). The average learning time τ increases for decreasing α, as $\tau \sim \alpha^{-0.89}$, whereas the best performance increases for slow plastic adaptations, as $\sim \alpha^{-0.017}$. Right: Universal learning curve obtained by rescaling the axes according to $S = \alpha^{-0.017} f(t/\tau)$. The scaling relations obtained for single rule learning are $\tau \sim \alpha^{-1}$ and $S = \alpha^{-0.05} f(t/\tau)$.

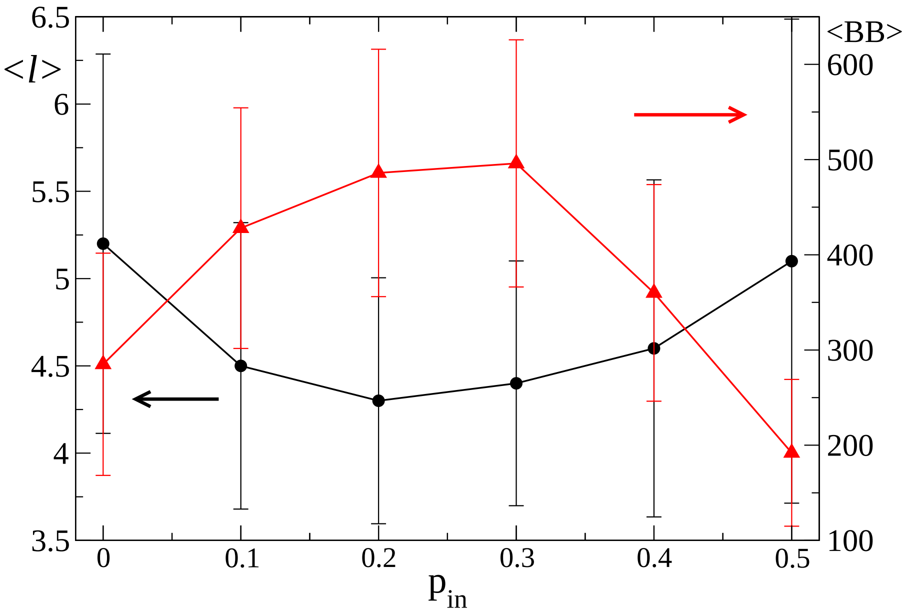

Supplementary Figure 4. The average number of neurons involved in avalanches giving the right answer to, both, the AND and XOR rules, $< BB >$, for 500 configurations of networks with $N = 1000$ neurons and different percentages of inhibitory synapses ($k_d = 3$, $\alpha = 0.001$). The maximum value is detected for p_{in} close to 20-30%. Correspondingly, the average shortest path, $< l >$, connecting input and output neurons exhibits a minimum value in this range.

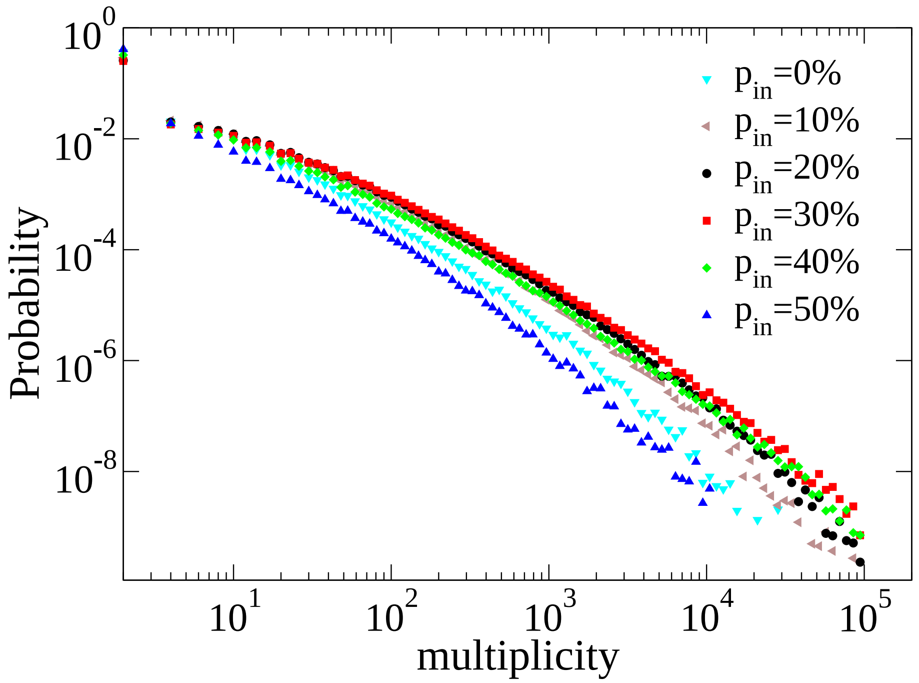

Supplementary Figure 5. Distribution of single neuron multiplicity, i.e., number of independent synaptic paths passing through a neuron, for backbones obtained by learning both the AND and XOR rules. Data are collected from 500 configurations of networks with $N = 1000$ neurons and different percentages of inhibitory synapses ($k_d = 3$, $\alpha = 0.001$). The behaviour of the distribution is non-monotonic with p_{in}: Larger values of the multiplicity are observed for 30% inhibitory synapses, suggesting that the backbone is organized in a more intricate structure of interconnected neurons for this fraction of inhibitory synapses.
